# Supplementary material for: A statistical framework for assessing pharmacological responses and biomarkers using uncertainty estimates
Source: eLife. 2020 Dec 4;9:e60352. doi: 10.7554/eLife.60352 (PMC7746236; doi:10.7554/eLife.60352)
Supplement: Transparent reporting form [file elife-60352-transrepform.docx]

***eLife’s* transparent reporting form**

We encourage authors to provide detailed information *within their submission* to facilitate the interpretation and replication of experiments. Authors can upload supporting documentation to indicate the use of appropriate reporting guidelines for health-related research (see [EQUATOR Network](http://www.equator-network.org/%20)), life science research (see the [BioSharing Information Resource](https://biosharing.org/)), or the [ARRIVE guidelines](http://www.plosbiology.org/article/info:doi/10.1371/journal.pbio.1000412) for reporting work involving animal research. Where applicable, authors should refer to any relevant reporting standards documents in this form.

If you have any questions, please consult our Journal Policies and/or contact us: [editorial@elifesciences.org](mailto:editorial@elifesciences.org).

**Sample-size estimation**

- You should state whether an appropriate sample size was computed when the study was being designed
- You should state the statistical method of sample size computation and any required assumptions
- If no explicit power analysis was used, you should describe how you decided what sample (replicate) size (number) to use

Please outline where this information can be found within the submission (e.g., sections or figure legends), or explain why this information doesn’t apply to your submission:

Our manuscript describes a novel methodology for estimating drug response, and applied this method to perform secondary data analysis of the Genomics of Drug Sensitivity in Cancer (GDSC) dataset. As this is secondary data analysis, and we did not design the original study, no sample size computation was required or performed. Detail on the design of the original study can be found in Iorio et al. (2016), as well as Garnett et al. (2012). We also included an analysis of a previously unpublished replicate dataset of cell sensitivity assays. No formal power calculation was performed to decide on the number of cell lines and replicates; rather, we measured the maximum number of cell lines and replicates that was achievable with the available resources and person-hours.

**Replicates**

- You should report how often each experiment was performed
- You should include a definition of biological versus technical replication
- The data obtained should be provided and sufficient information should be provided to indicate the number of independent biological and/or technical replicates
- If you encountered any outliers, you should describe how these were handled
- Criteria for exclusion/inclusion of data should be clearly stated
- High-throughput sequence data should be uploaded before submission, with a private link for reviewers provided (these are available from both GEO and ArrayExpress)

Please outline where this information can be found within the submission (e.g., sections or figure legends), or explain why this information doesn’t apply to your submission:

Information about replicates can be found in the Results section of the manuscript. Different cell lines of the same cancer type constitute biological replicates, while technical replicates were only available for the replication experiment; we believe this is clear from the manuscript. In cases where outliers have been omitted from plots, this has been described in the figure captions.

**Statistical reporting**

- Statistical analysis methods should be described and justified
- Raw data should be presented in figures whenever informative to do so (typically when N per group is less than 10)
- For each experiment, you should identify the statistical tests used, exact values of N, definitions of center, methods of multiple test correction, and dispersion and precision measures (e.g., mean, median, SD, SEM, confidence intervals; and, for the major substantive results, a measure of effect size (e.g., Pearson's r, Cohen's d)
- Report exact p-values wherever possible alongside the summary statistics and 95% confidence intervals. These should be reported for all key questions and not only when the p-value is less than 0.05.

Please outline where this information can be found within the submission (e.g., sections or figure legends), or explain why this information doesn’t apply to your submission:

We have clearly described the statistical tests and summary statistics in the Results section of the manuscript. There we have clearly stated the statistical tests used, multiple testing correction, appropriate measures of dispersion and precision, and sample sizes. Additionally, our statistical approach is clearly described in the Methods section of the manuscript and open source code for our approach can be found on GitHub (<https://github.com/FrankD/GPDrugModels>).

(For large datasets, or papers with a very large number of statistical tests, you may upload a single table file with tests, Ns, etc., with reference to sections in the manuscript.)

**Group allocation**

- Indicate how samples were allocated into experimental groups (in the case of clinical studies, please specify allocation to treatment method); if randomization was used, please also state if restricted randomization was applied
- Indicate if masking was used during group allocation, data collection and/or data analysis

Please outline where this information can be found within the submission (e.g., sections or figure legends), or explain why this information doesn’t apply to your submission:

As the main analysis in the paper is a secondary data analysis, we were not involved in the original study design. Matched control groups and random allocation of cell lines were used in the drug screening experiments that generated the data we used. Detail on the design of the drug response experiments can be found in Iorio et al. (2016), as well as Garnett et al. (2012).

**Additional data files (“source data”)**

- We encourage you to upload relevant additional data files, such as numerical data that are represented as a graph in a figure, or as a summary table
- Where provided, these should be in the most useful format, and they can be uploaded as “Source data” files linked to a main figure or table
- Include model definition files including the full list of parameters used
- Include code used for data analysis (e.g., R, MatLab)
- Avoid stating that data files are “available upon request”

Please indicate the figures or tables for which source data files have been provided:

We have released the code for our curve fitting method and Bayesian biomarker detection on GitHub (<https://github.com/FrankD/GPDrugModels>). We have supplied the required source data to reproduce our results as the following supplementary tables.

1. Supplementary Table S1 - Summary of pharmacogenomic associations based on ANOVA

2. Supplementary Table S2 - Pharmacogenomic associations based on Bayesian testing of GP curve fits

3. Supplementary Table S3 - Raw and curve fitted replicate dataset

4. Supplementary Table S4 - GP curve fits dataset with calculated summary statistics
